# An Autophagy-Related Gene Signature Associated With Clinical Prognosis and Immune Microenvironment in Gliomas

**DOI:** 10.3389/fonc.2020.571189

**Published:** 2020-10-19

**Authors:** Yang Xu, Renpeng Li, Xiaoxia Li, Naijun Dong, Di Wu, Lin Hou, Kan Yin, Chunhua Zhao

**Affiliations:** ^1^School of Basic Medicine, Qingdao University, Qingdao, China; ^2^Department of Neurosurgery, Beijing Tiantan Hospital, Capital Medical University, Beijing, China

**Keywords:** glioma, autophagy-related genes, prognosis, signature, tumor immune environment

## Abstract

Glioma is one of the leading causes of death from cancer, and autophagy-related genes (ARGs) play an important role in glioma occurrence, progression, and treatment. In this study, the gene expression profiles and clinical data of glioma patients were obtained from The Cancer Genome Atlas (TCGA) and the Chinese Glioma Genome Atlas (CGGA), respectively. ARGs were obtained from the Human Autophagy Database. We analyzed the expression of the ARGs in glioma and found that 73 ARGs were differentially expressed in tumor and normal tissues. Univariate Cox regression analysis was used to identify prognostic differentially expressed ARGs (PDEARGs). Least absolute shrinkage and selection operator (LASSO) and multivariate Cox regression analyses were performed on the PDEARGs to determine the risk genes; and BRIC5, NFE2L2, GABARAP, IKBKE, BID, MAPK3, FKBP1B, MAPK8IP1, PRKCQ, CX3CL1, NPC1, HSP90AB1, DAPK2, SUPT20H, and PTEN were selected to establish a prognostic risk score model for TCGA and CGGA cohorts. This model accurately stratified patients with different survival outcomes, and the autophagy-related signature was also appraised as being an independent prognostic factor. We also constructed a prognostic nomogram using risk score, age, gender, WHO grade, isocitrate dehydrogenase (IDH) mutation status, and 1p/19q co-deletion status; and the calibration plots showed excellent prognostic performance. Finally, Pearson correlation analysis suggested that the ARG signature also played an essential role in the tumor immune microenvironment. In summary, we constructed and verified a novel autophagy-related signature that was tightly associated with the tumor immune microenvironment and could serve as an independent prognostic biomarker in gliomas.

## Introduction

Glioma is one of the most common primary brain tumors in adults, accounting for about 15% of all brain tumors and more than 48% of malignant brain tumors (1). The prognosis of patients with glioma is poor, despite the use of multi-mode therapy, including neurosurgery, radiotherapy, chemotherapy, targeted therapy, and immunotherapy, due to the high recurrence and mortality rates (2, 3). For glioma patients, it still appears difficult to predict and extend survival, and the identification of novel prognostic biomarkers for glioma is therefore imperative. Autophagy is the process of degradation and circulation of proteins and intracellular components during starvation or stress (4). It occurs at the basal level of all cells and is fundamental to cell homeostasis (5, 6). Autophagy often plays a key role in the pathogenesis of diseases, such as type 2 diabetes, cardiovascular disease, neurological disease, and cancer (7). Autophagy also has a dual function in tumors, eliminating damaged proteins and organelles in the early stages of the tumor, reducing cell damage, and thus inhibiting tumor development (8). However, once tumors have formed, autophagy via HIF1α/AMPK can provide nutrients to promote tumor cell growth when nutrients are limited (9). In most cases, autophagy is thought to inhibit early tumorigenesis but promote the development of established tumors.

To illustrate the importance of autophagy, there is increasing evidence of the role of autophagy-related genes (ARGs) in the development of cancer. High expression of Atg10 is associated with poor prognosis for lung cancer (10), and autophagy gene Atg7 knockout leads to the progression of lung cancer to eosinophilic cell tumors, inhibiting tumor proliferation (11). Autophagy has been negatively correlated with mTOR substrate phosphorylation, and recent studies have shown that high expression of LC3/beclin 1 is associated with poor prognosis in glioma patients (12). In addition, autophagy-related proteins, such as cathepsin D and LAMP2, are overexpressed in glioma and may also be potential targets for prognosis and prediction (13). These findings confirm the role of autophagy in cancer and suggest that ARGs may serve as prognostic markers for glioma.

Although some previous studies have explored the relationship between ARGs and prognosis in glioma patients, those studies have focused on the function of a single gene. Very few studies have used gene expression profiles to examine the relationship between multiple ARGs and glioma prognosis. Therefore, in this study, we develop a reliable prognostic model based on ARGs and explore the clinical application of the ARG signatures in glioma patients.

## Materials and Methods

### Acquisition of Glioma Datasets

The RNA-Seq and somatic mutation data of 703 patients were downloaded from The Cancer Genome Atlas (TCGA) data portal (14)^1^ and used as a training cohort. From the Chinese Glioma Genome Atlas (CGGA) dataset^2^, we collected the RNA-Seq data of 1,018 samples as a validation cohort (15, 16). We compared transcriptome data with the patients’ clinical information based on the patient ID numbers. If a patient’s ID number did not match, we removed it. Thus, 665 patients with complete overall survival (OS) information and gene expression profiles were obtained from TCGA database, while 929 patients with complete OS information and gene expression profiles were obtained from the CGGA database. The 232 ARGs were obtained from the human autophagy HADb database^3^ (17). Immune infiltrate data for glioma patients were derived through a deconvolution algorithm based on a normalized gene expression profile (CIBERSORT), which was used to characterize the infiltrating components of the immune cells in 22 complex samples (18, 19).

### Identification of Differentially Expressed Autophagy-Related Genes

The R package “edgeR” was used to screen differentially expressed genes (DEGs) in glioma and normal neural tissue samples (20). A Wilcoxon signed-rank test was used to screen DEGs according to the cutoff values’ false discovery rate (FDR) <0.05 and |log2 FC| > 1. The intersection of DEGs and ARGs was considered to be the set of significant differentially expressed ARGs (DEARGs).

### Construction and Verification of a Prognostic Risk Score Model Based on Autophagy-Related Genes

We chose the data in TCGA dataset as the training cohort, which was used to construct the Cox proportional hazard regression model, and the data in the CGGA dataset was used as the validation cohort, which was used to validate the model’s performance. First, univariate Cox analysis was used to identify possible prognostic DEARGs (PDEARGs) by using the “survival” package with a *P-*value of <0.05 (21). Second, the potential risk genes were selected, and the overfitting genes were eliminated by least absolute shrinkage and selection operator (LASSO) regression using the “glmnet” R package (22). Third, Cox proportional hazard regression was used to construct the prognostic risk model by using the “glmnet” R package (22). The following formula was used to establish the prognostic risk model:

$$r\,i\,s\,k\,s\,c\,o\,r\,e=\sum_{j=1}^{n}\left(C\,o\,e\,f_{j}\times X_{j}\right),$$

where *Coef* is the multivariate Cox regression analysis coefficient of the ARGs and *X* is the relative expression level of each ARG. The patients were divided into high-risk and low-risk groups, with the median risk score used as the cutoff value and high-risk scores suggesting that the prognosis of the patients was poor.

### Statistical Analysis

R software (3.6.1) was used for all statistical analyses. Pearson correlation coefficients were used to evaluate the rank correlations among the variables. Independent *t*-tests were used to assess differences between the variables. Kaplan–Meier curve and log-rank tests were used for survival data analysis, and univariate Cox regression analysis was used for survival factor analysis. Multivariate Cox regression analysis was used to identify independent prognostic factors. The accuracy of the prognostic model was assessed by time-dependent receiver operating characteristic (ROC) analysis. A *P*-value of <0.05 was considered statistically significant.

## Results

### Identification of Differentially Expressed Autophagy-Related Gene and Functional Enrichment Analysis

**FIGURE 1:**
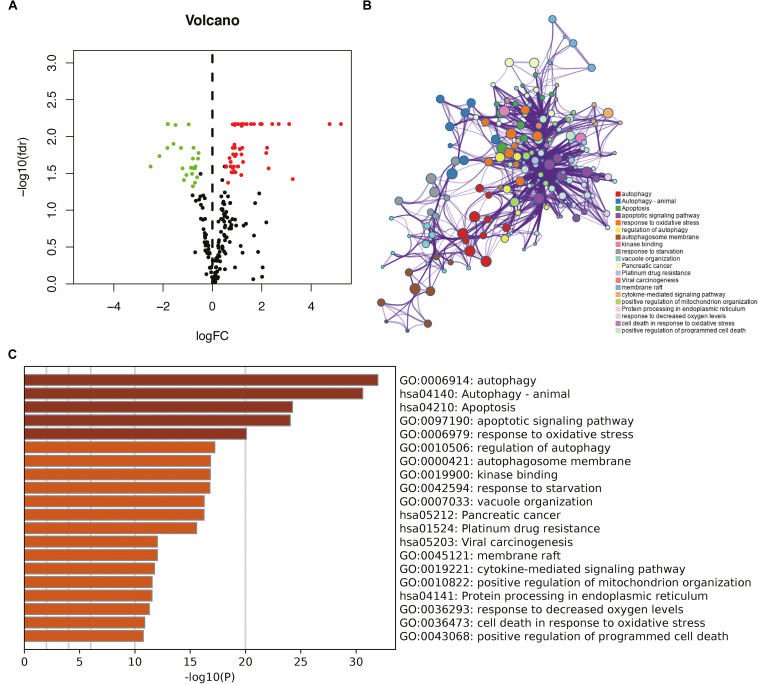
Identification of differentially expressed autophagy-related genes (DEARGs) in glioma and biological function analysis. **(A)** Volcano plot of DEARGs in tumor and normal samples of The Cancer Genome Atlas (TCGA) dataset; the green dots represent down-regulated genes, and red dots represent up-regulated genes. **(B)** The functional enrichment map of pathways. Each node indicates an enriched term and is colored by its cluster ID. **(C)** Enriched pathways of the genes positively correlated with DEARGs.

The mRNA levels of genes in gliomas (*n* = 698) and brain tissues (*n* = 5) in TCGA cohort were examined, and these values were compared using the Wilcoxon signed-rank test. This analysis revealed 15,001 DEGs in glioma tissues compared with normal brain tissues [FDR < 0.05, |log2 fold-change (FC)| > 1]. Then, we obtained 73 DEARGs in glioma tissues compared with normal brain tissues after taking the intersection of DEGs and ARGs (Figure 1A).

To explore the potential functional characteristics of the DEARGs, we annotated their functions using Metascape. The results showed that these genes were mainly involved in autophagy, animal autophagy, apoptosis, apoptotic signaling pathways, and the response to oxidative stress (Figures 1B,C).

### Identification of Prognostic Differentially Expressed Autophagy-Related Genes

To identify the PDEARGs, we performed a univariate Cox regression analysis of each DEARG expression in TCGA cohort. A total of 62 DEARGs were significantly correlated with OS in glioma patients (*P* < 0.05).

### Training Cohort to Identify Risk Genes for Inclusion in the Risk Model

**FIGURE 2:**
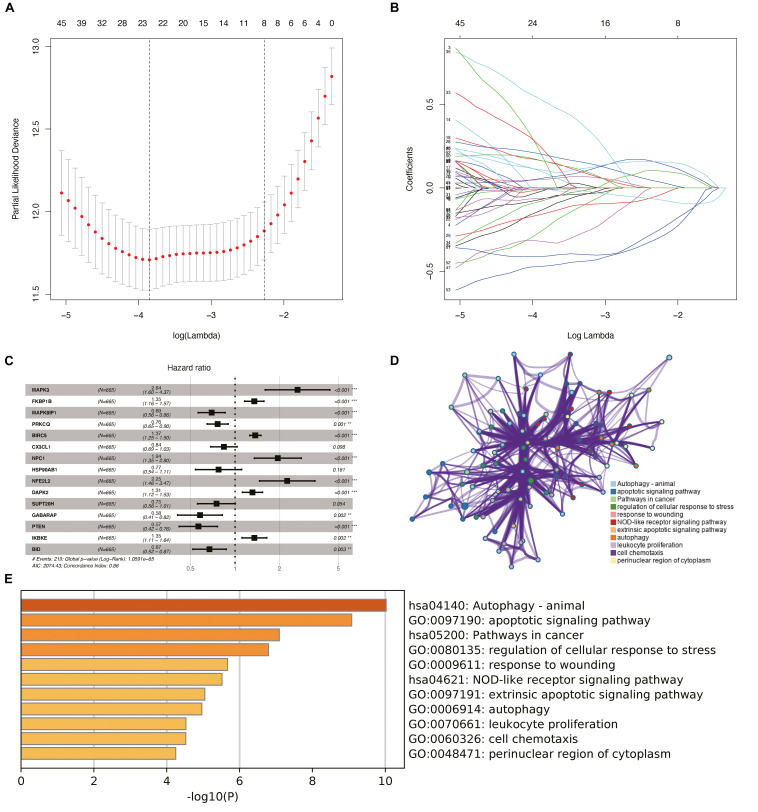
Construction of the prognostic risk model and biological function analysis. **(A)** Cross-validation for tuning parameter screening in the least absolute shrinkage and selection operator (LASSO) regression model. **(B)** LASSO coefficient profiles of the common genes. **(C)** Risk genes in the prognostic risk model. **(D)** The functional enrichment map of pathways of risk genes. Each node indicates an enriched term and is colored by its cluster ID. **(E)** Enriched pathways of the genes positively correlated with risk genes.

Considering the effect of PDEARGs on patient prognosis, we further screened the PDEARGs by constructing a Cox regression hazard model, selecting the potential risk genes, and eliminating the overfitting genes in the model by LASSO regression; 23 genes (Figures 2A,B) were thus obtained and further analyzed by multivariate Cox proportional risk regression analysis. Finally, 15 risk genes (BRIC5, NFE2L2, GABARAP, IKBKE, BID, MAPK3, FKBP1B, MAPK8IP1, PRKCQ, CX3CL1, NPC1, HSP90AB1, DAPK2, SUPT20H, and PTEN) were identified, and the risk scores for each case were calculated using their expression levels and regression coefficients (Figure 2C). Among these genes, FKBP1B, BIRC5, NFE2L2, and IKBKE were considered high-risk genes (poor prognosis), while MAPK3, NPC1, DAPK2, MAPK8IP1, PRKCQ, CX3CL1, HSP90AB1, SUPT20H, GABARAP, PTEN, and BID were considered low-risk genes (protective factors) for patients’ OS. To explore the potential functional characteristics of the risk genes, we annotated their functions using Metascape. The results showed that these genes are mainly involved in animal autophagy, apoptotic signaling pathways, pathways in cancer, and the regulation of cellular response to stress (Figures 2D,E).

We then used clinical specimens from the Human Protein Atlas^4^ to analyze the expression of the proteins encoded by the risk genes (no data were found for DAPK2, PRKCQ, MAPK8IP1, and GABARAP). MAPK3 and CX3CL1 were strongly positive; NFE2L2, HSP90AB1, and SUPT20H were moderately positive; and FKBP1B, BIRC5, NPC1, IKBKE, BID, and PTEN were weakly positive in glioma tissue relative to their expression levels in normal tissue (Supplementary Figures S1A–K). Kaplan–Meier survival curves were also constructed to evaluate the relationship between the expression levels of the prognostic genes and OS, and the results showed that the low-expression groups of FKBP1B, BIRC5, NFE2L2, and IKBKE (*P* < 0.05) had better prognosis (Supplementary Figures S2B,C,E,G) and that the high-expression groups of MAPK3, NPC1, DAPK2, MAPK8IP1, PRKCQ, CX3CL1, HSP90AB1, SUPT20H, GABARAP, PTEN, and BID (*P* < 0.05) had better prognosis (Supplementary Figures S2A,D,F,H–O).

### Construction and Verification of the Prognostic Risk Model

To assess the significance of the risk genes in the prognosis of patients with glioma in the risk models, we used estimated regression coefficients and the expression levels of the risk genes to calculate the risk scores for each patient. The calculation formula is as follows:

$$\begin{array}{c} \text{Risk}\;\text{score=}(\text{0}.\text{972364596}\times \text{MAPK3}\;\text{expression})\\ \text{+}(\text{0}.\text{299641268}\times \text{FKBP1B}\;\text{expression})\\ \text{+}(\text{-0}.\text{364718845}\times \text{MAPK8IP1}\;\text{expression})\\ \text{+}(\text{-0}.\text{271491791}\times \text{PRKCQ}\;\text{expression})\\ \text{+}(\text{0}.\text{314270977}\times \text{BIRC5}\;\text{expression})\\ \text{+}(\text{-0}.\text{172912776}\times \text{CX3CL1}\;\text{expression})\\ \text{+}(\text{0}.\text{664977132}\times \text{NPC1}\;\text{expression})\\ \text{+}(\text{-0}.\text{257268535}\times \text{HSP90AB1}\;\text{expression})\\ \text{+}(\text{0}.\text{81239816}\times \text{NFE2L2}\;\text{expression})\\ \text{+}(\text{0}.\text{271699944}\times \text{DAPK2}\;\text{expression})\\ \text{+}(\text{-0}.\text{289118873}\times \text{SUPT20H}\;\text{expression})\\ \text{+}(\text{-0}.\text{545908599}\times \text{GABARAP}\;\text{expression})\\ \text{+}(\text{-0}.\text{570898637}\times \text{PTEN}\;\text{expression})\\ \text{+}(\text{0}.\text{300429145}\times \text{IKBKE}\;\text{expression})\\ \text{+}(\text{-0}.\text{398268499}\times \text{BID}\;\text{expression}). \end{array}$$

**FIGURE 3:**
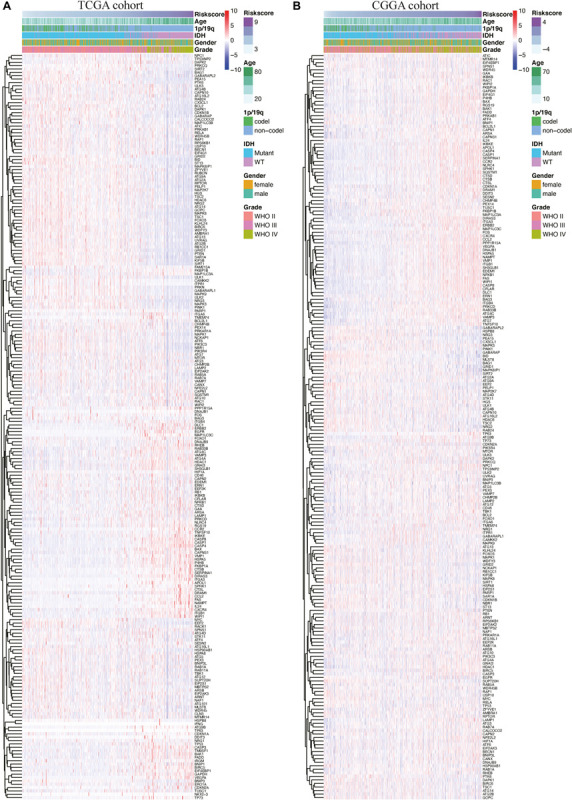
Heatmaps of autophagy-related genes based on the risk score value in The Cancer Genome Atlas (TCGA) cohort **(A)** and the Chinese Glioma Genome Atlas Chinese Glioma Genome Atlas (CGGA) cohort **(B)**.

In the CGGA cohort, the risk score was calculated using the same risk genes and regression coefficients for validation. The heatmaps show an overview of the correlation between ARG expression and clinical characteristics in TCGA and CGGA cohorts (Figure 3).

**FIGURE 4:**
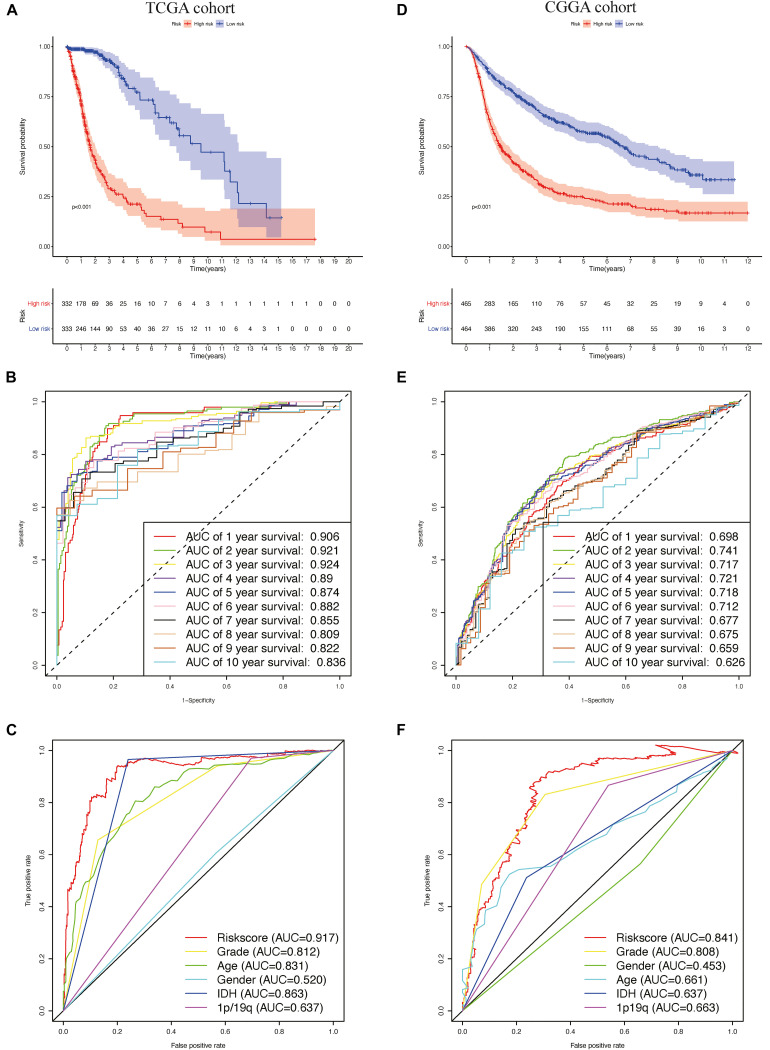
The prognostic value of the risk signature in The Cancer Genome Atlas (TCGA) and the Chinese Glioma Genome Atlas (CGGA) cohorts. **(A)** Kaplan–Meier (K–M) survival analysis of the risk score in TCGA cohort. **(B,C)** Time-dependent receiver operating characteristic (ROC) curve analysis of the risk score and traditional factors in TCGA cohort. **(D)** Kaplan–Meier (K–M) survival analysis of the risk signature in the CGGA cohort. **(E,F)** Time-dependent ROC curve analysis of the risk score and traditional factors in the CGGA cohort.

Patients in TCGA cohort were divided into a high-risk group (*n* = 332) and a low-risk group (*n* = 333), according to the median risk score. We used a Kaplan–Meier curve based on the log-rank test to identify the difference in prognosis between the high-risk group and the low-risk group. The prognosis of patients in the high-risk group was worse than that in the low-risk group (*P* < 0.05) (Figure 4A).

**FIGURE 5:**
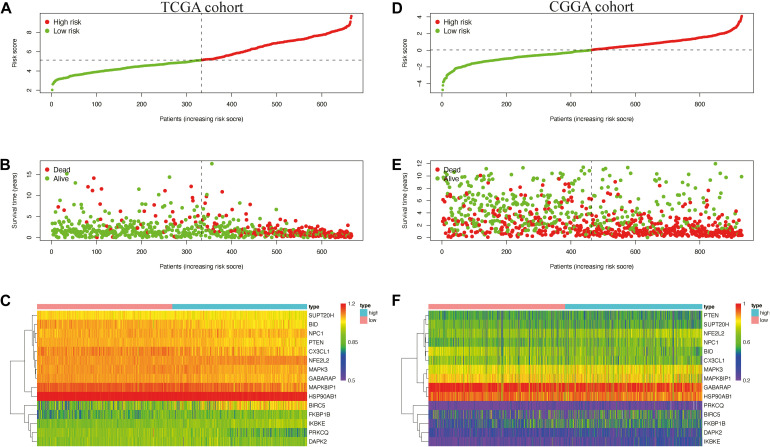
Distribution of the risk score **(A,D)** survival status **(B,E)** and expression level of risk genes **(C,F)** in the Cancer Genome Atlas (TCGA) and the Chinese Glioma Genome Atlas (CGGA) cohort.

We then used time-dependent ROC curves to confirm the accuracy of the model’s predictions for 1–10 years. The ROC (AUC) of the prediction model was 0.906 over 1 year, 0.921 over 2 years, 0.924 over 3 years, 0.89 over 4 years, 0.874 over 5 years, 0.882 over 6 years, 0.855 over 7 years, 0.809 over 8 years, 0.822 over 9 years, and 0.836 over 10 years in TCGA training cohort (Figure 4B), all of which were significantly higher than the other factors (Figure 4C), emphasizing the superior predictive value of the prognostic risk model. We then ranked the risk scores of the patients in TCGA cohort and analyzed their distribution (Figure 5A). The survival status of each patient in TCGA cohort is shown in Figure 5B.

We also generated heatmap to describe the expression patterns of risk genes in both the high-risk and low-risk groups (Figure 5C). In patients with high-risk scores in TCGA cohort, the high-risk genes (BIRC5, FKBP1B, IKBKE, and NFE2L2) were up-regulated, while the protective genes (BID, CX3CL1, GABARAP, HSP90AB1, MAPK3, MAPK8IP1, NPC1, PRKCQ, PTEN, and SUPT20H) were down-regulated. In patients with low risk scores, these risk genes showed opposite patterns of expression.

To validate the accuracy of the prognostic risk model, the patients in the CGGA cohort were also divided into two groups based on the median risk score. Next, we used Kaplan–Meier survival analysis to identify the prognostic differences between the high-risk group and the low-risk group. The Kaplan–Meier survival curves differed significantly between the two risk groups in the CGGA cohort (*P* < 0.05) (Figure 4D), with the patients’ survival rates being higher in the low-risk group than in the high-risk group throughout the follow-up period. ROC analyses were also performed for the CGGA cohort over 1–10 years; the AUCs for each year were, in order, 0.698, 0.741, 0.717, 0.721, 0.718, 0.712, 0.677, 0.675, 0.659, and 0.626 (Figure 4E), which were higher than for the traditional factors (Figure 4F). The risk score distribution, survival status, and risk gene expression in the CGGA cohort are shown in Figures 5D,E. Similar to the results in TCGA cohort, the most protective gene levels were higher and the most risk gene levels lower in the low-risk group than in the high-risk group (Figure 5F). These results indicate that the prognostic risk model is capable of precisely predicting the prognosis of glioma patients.

In addition, when the patients were stratified according to different clinicopathologic parameters, the autophagy signature remained a significant prognostic factor in both TCGA cohort (Supplementary Figures S3A–J) and the CGGA cohort (Supplementary Figures S3K–T).

### Independent Prognostic Value of the Prognostic Risk Model

**FIGURE 6:**
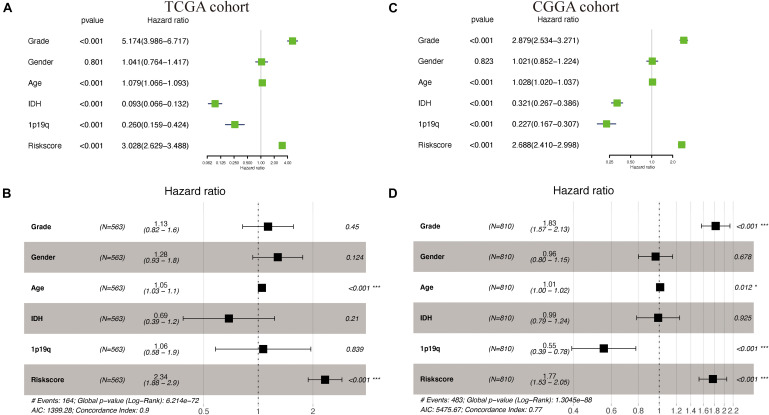
Cox regression analyses in The Cancer Genome Atlas (TCGA) and the Chinese Glioma Genome Atlas (CGGA) cohorts. **(A,B)** Univariate and multivariate Cox regression analyses of risk score and pathologic characteristics in TCGA cohort. **(C,D)** Univariate and multivariate Cox regression analyses of risk score and pathologic characteristics in the CGGA cohort.

Supplementary Table S1 shows the demographics and clinicopathologic characteristics of glioma patients in TCGA cohort and CGGA cohorts based on the autophagy signature. Univariate and multivariate Cox regression analyses were then performed to assess whether our model-generated risk scores were independent of other clinical parameters [WHO grade, age, gender, isocitrate dehydrogenase (IDH) mutation status, 1p/19q co-deletion status, and risk score] as prognostic factors for patients with glioma. Univariate analysis showed that WHO grade, age, IDH mutant status, 1p/19q co-deletion status, and risk score were associated with the prognosis of glioma patients in both TCGA and CGGA cohorts (*P* < 0.05) (Figures 6A,C); only gender was not. Multivariate analysis showed that age and risk score were independently associated with OS in TCGA cohort (*P* < 0.05), while WHO grade, 1p/19q co-deletion status, and risk score were independently associated with OS in the CGGA cohort (*P* < 0.05) (Figures 6B,D). These results suggest that the prognostic risk model can be independently used to predict the prognosis of patients with glioma.

### Construction and Validation of the Nomogram

**FIGURE 7:**
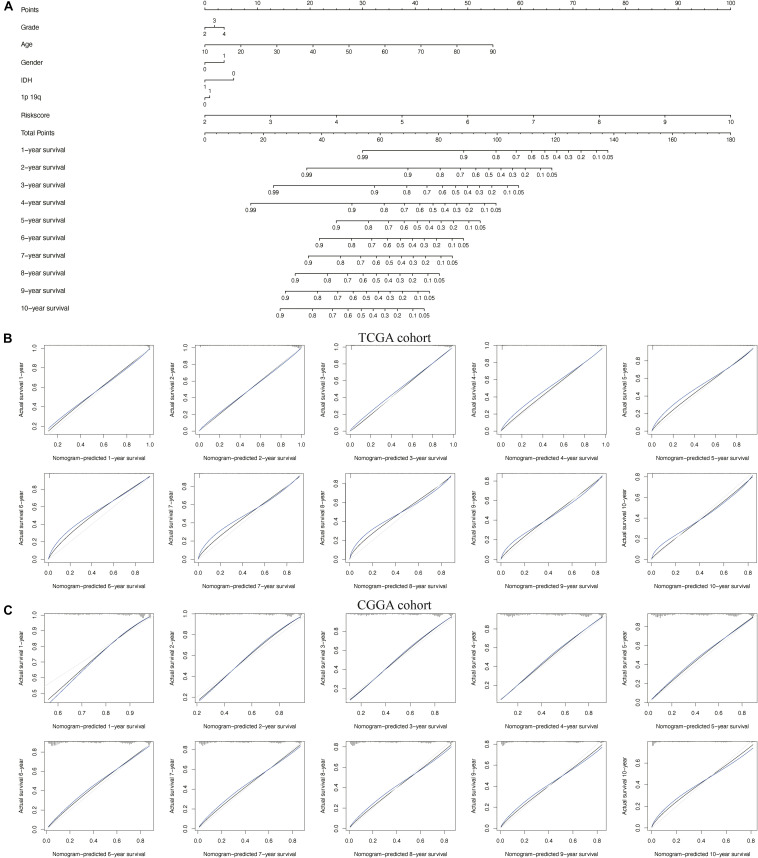
Nomogram for predicting overall survival of patients with glioma. **(A)** A nomogram integrating the signature risk score with the pathologic characteristics. **(B,C)** The calibration curve for the nomogram in The Cancer Genome Atlas (TCGA) and Chinese Glioma Genome Atlas (CGGA) cohorts.

To better predict the prognosis of patients with glioma at 1–10 years, we constructed a new nomogram (Figure 7A). In Figures 7B,C, the blue lines represent the observed survival rate, the gray lines represent the ideal survival rate, and the black lines represent the optimized modified survival rate. The optimism-corrected line, also known as the bias-corrected or overfitting-corrected line, was produced using a bootstrap approach to estimate the observed and predicted values based on a non-parametric smoother applied to a sequence of predicted values (23). For the predicted 1- to 10-year survival plots in the training cohort (Figure 7B) and the validation cohort (Figure 7C), the observed and optimism-corrected lines are aligned, although the two are slightly different from the ideal 45° line, which means that the 1- to 10-year survival predicted by the nomogram reflects the observed actual survival in both the training and validation cohorts.

### Clinical Utility of the Prognostic Risk Model

**FIGURE 8:**
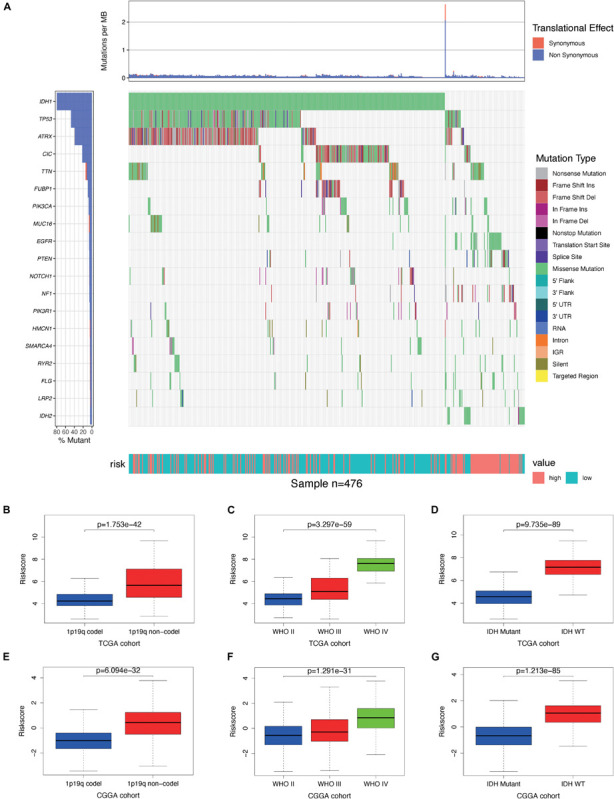
Association between genomic alterations, pathologic characteristics, and the autophagy-related signature. **(A)** Differential somatic mutation analysis between high-risk group and low-risk group in The Cancer Genome Atlas (TCGA) cohort. **(B–D)** Distribution of the risk score in glioma patients stratified by 1p/19q codeletion status, WHO grade, and isocitrate dehydrogenase (IDH) mutation status in TCGA cohort. **(E–G)** Distribution of the risk score in glioma patients stratified by 1p/19q codeletion status, WHO grade, and IDH mutation status in the Chinese Glioma Genome Atlas (CGGA) cohort.

By analyzing somatic mutation data from TCGA dataset, we explored differences in genomic changes between the high-risk and low-risk groups, as some genomic changes have a negative impact on glioma survival (24). By comparing the frequency of mutation occurrences, we found that there was some significant mutation enrichment between the two groups – IDH1, TP53, ATRX, and CIC mutations were more abundant in the low-risk group (Figure 8A). By examining the association between risk scores and clinical features in both TCGA and CGGA cohorts, we found that the risk score was positively correlated with the grade of glioma (Figures 8C,F) and that it was significantly lower in the 1p/19q co-deletion and IDH mutation samples (Figures 8B,D,E,G).

To examine the performance of our model in predicting the progression of glioma, we analyzed the relationships between the clinical variables in the two cohorts and the risk genes. We found that the expressions of BRIC5, FKBP1B, IKBKE, and NFE2L2 were significantly greater in the 1p/19q non-co-deletion samples, while the expressions of BID, CX3CL1, DAPK2, GABARAP, HSP90AB1, MAPK3, MAPK8IP1, and SUPT20H were significantly lower in those samples in both cohorts (Supplementary Figure S4); the expressions of BRIC5, FKBP1B, IKBKE, and NFE2L2 were positively correlated, and the expressions of BID, CX3CL1, DAPK2, MAPK3, MAPK8IP1, and PRKCQ negatively correlated with the grade of glioma in both cohorts (Supplementary Figure S5); and the expressions of BRIC5, FKBP1B, IKBKE, and NFE2L2 were significantly greater in IDH wild-type samples, while the expressions of BID, CX3CL1, GABARAP, HSP90AB1, MAPK3, MAPK8IP1, PRKCQ, and SUPT20H were significantly lower in those samples in both cohorts (Supplementary Figure S6) (*P* < 0.05). These results demonstrate that the dysregulation of autophagy-related risk gene expression is associated with the development of glioma.

**FIGURE 9:**
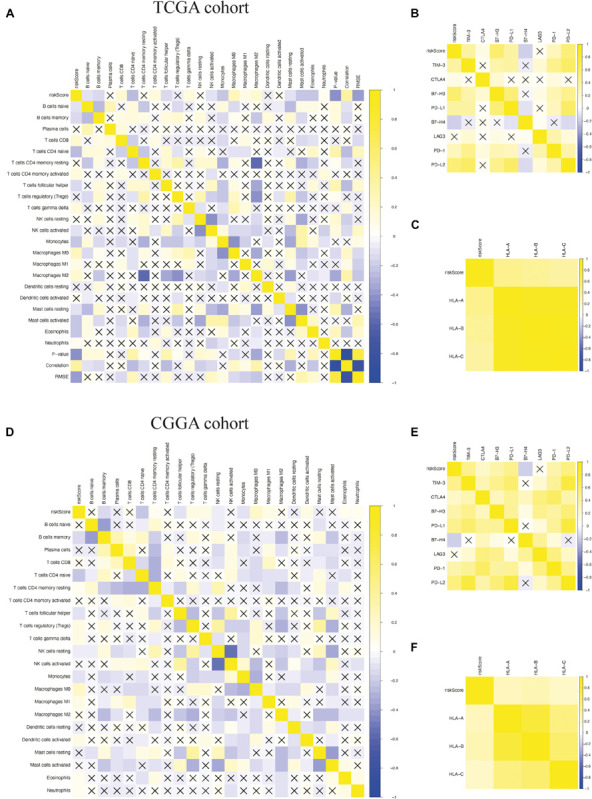
Pearson correlation analysis between the risk score and immune cell infiltration, immune checkpoints, and human leukocyte antigen (HLA) complex expressions. **(A–C)** Pearson correlation analysis between the risk score and the level of immune cell infiltration, the expressions of immune checkpoints, and the expressions of HLA complex in The Cancer Genome Atlas (TCGA) cohort. **(D–F)** Pearson correlation analysis between the risk score and the level of immune cell infiltration, the expressions of immune checkpoints, and the expressions of HLA complex in the Chinese Glioma Genome Atlas (CGGA) cohort. “X” means *P* > 0.05.

To determine whether the model could reflect the status of the tumor immune microenvironment in glioma patients, we analyzed the correlation between the risk scores and immune cell infiltration by using Pearson correlation analysis in both TCGA and CGGA cohorts. We found that the risk scores were correlated positively with the levels of CD8+ T cells; gamma delta T cells; M0, M1, and M2 macrophages; and activated dendritic cells. Meanwhile, they were negatively correlated with the levels of naive B cells, naive CD4+ T cells, follicular helper T cells, activated NK cells, monocytes, and resting mast cells, suggesting that the immune-related gene signature could indicate the level of infiltrating immune cells in gliomas to a certain extent (Figures 9A,D). We also found that risk scores were correlated positively with the expressions of the immune checkpoint (B7-H3, CTLA4, LAG3, PD-1, PD-L1, PD-L2, and TIM-3) (Figures 9B,E) and inflammatory factors (HLA-A, HLA-B, and HLA-C) by using Pearson correlation analysis in TCGA and CGGA cohorts (Figures 9C,F), indicating that the risk scores are also positively associated with an elevated level of immune exhaustion and that the immune-related gene signature might promote activation of inflammatory responses in glioma patients. In conclusion, the immune-related gene signature could promote the malignant progression of glioma by shaping the tumor immune microenvironment.

## Discussion

Glioma is a progressive disease that requires an effective prognostic indicator for diagnosis and treatment. There is increasing evidence that autophagy plays a key role in the occurrence, progression, and therapeutic resistance of glioma (4, 8, 25). And previous studies have shown that autophagy inhibits its development. In the KRAS-driven glioblastoma mouse model, autophagy blocked Atg7, Atg13, or Ulk1 by shRNA; inhibited the occurrence and growth of tumors; and extended the survival of mice (26). Chio et al. reported that treatment of temozolomide (TMZ)-resistant human glioma cells with honokiol can reduce cell activity and induce apoptosis, and Zhang et al. (27) showed that ARG MAPK8IP1 overexpression and SH3GLB1 knockdown inhibited glioma cell progression and improved TMZ sensitivity. There have also been many studies on targeted autophagy therapy for glioma in recent years; for example, 2-propylpentanoic acid (VPA) combined with TMZ has been found to have significant antitumor effects on TMZ-resistant malignant glioma cells by inducing autophagy (28). ARGs are therefore a promising therapeutic target and prognostic predictor in glioma.

In this study, we identified prognostic ARGs and used them to construct a reliable model for predicting the OS of patients with glioma. First, we analyzed the expression of 232 ARGs in glioma and found that the expression of 62 DEARGs was related to OS. These results suggest that ARGs are an important prognostic factor for glioma patients.

Next, we evaluated these PDEARGs in the stratification of patient outcomes. Through a combination of LASSO regression and Cox regression analysis, we identified 15 PDEARGs of interest and used them to construct a Cox regression risk model. We also analyzed and verified the reliability and stability of the model. The results showed that the model can accurately discriminate patients with different survival outcomes. Univariate and multivariate Cox regression analyses indicated that the prognostic risk model could independently predict the prognosis of patients with glioma. In addition, nomogram analysis indicated that the accuracy of the model in predicting glioma prognosis could be improved in combination with other clinical features.

Nomograms have been widely used in clinical work for visual presentation (29, 30). In this study, we created a nomogram containing WHO grade, age, gender, IDH mutation status, 1p/19q co-deletion status, and risk score. The calibration diagram based on the training and validation cohorts showed that the actual survival rates of patients with glioma were very close to the predicted survival rates, indicating that the prediction performance of the nomogram was excellent. The visual scoring system could help doctors and patients to make more personalized survival predictions, which could help them to choose better treatment options.

The relationship of risk genes with clinical variables (WHO grade, IDH mutation status, and 1p/19q codeletion status) was also analyzed. We found that risk genes were associated with the progression of gliomas. Our model therefore has high clinical application value in predicting the development of glioma.

A large number of studies have examined the molecular biomarkers of glioma using advances in large-scale genome sequencing technology (31–33). BIRC5 is a member of the family of genes that inhibit apoptosis, and, in glioma, BIRC5 expression is positively correlated with tumor grade (34, 35) – a recent study revealed that BIRC5 affects the tumorigenicity of glioma cells by regulating p53 protein (36). IKBKE is involved in the malignant transformation and development of tumors as an oncogene (37). Lu et al., have suggested that IKBKE plays a pivotal role in regulating cell proliferation and invasion and the epithelial–mesenchymal transition of malignant glioma cells *in vitro* and *in vivo* by impacting the Hippo pathway (38). The overexpression of IKBKE is closely related to the stage of the glioma, and the invasion and migration of IKBKE cells are reduced after IKBKE is silenced with synthetic siRNAs (39). The tumor-promoting activity of NFE2L2 has been attributed to its own function; its activity is generally enhanced in glioblastoma cell lines and tumors, and low NFE2L2 expression inhibits proliferation and self-renewal of glioma stem cells (40, 41). These results are consistent with our conclusions that the expressions of BIRC5, IKBKE, and NFE2L2 are positively correlated with the progression of glioma.

In addition to BRIC5, IKBKE, and NFE2L2, the 15 identified genes include many related to invasion and metastasis, such as PRKCQ, MAPK3, and PTEN. PRKCQ is a member of protein kinase C (PKC), and Couldwell et al., found a correlation between PKC activity and glioma cell proliferation, as glioma cells with a fast development speed have high PCK activity (42). MAPK3 is an important signal transduction molecule in the ERK/MAPK pathway, and previous studies have found that the enhanced functional activity of MAPK3 plays a critical role in the development and progression of gastric cancer (43, 44). Wang et al. have also investigated how MAPK3 regulates apoptosis and invasion in gliomas through targeting by mir-483-5p (45). PTEN dysregulation in mice leads to the development of multiple tumors (46, 47), and it has been reported that the up-regulation of PTEN expression is related to the growth inhibition of glioma cells *in vitro* (48).

Previous studies have demonstrated that immune infiltration plays a crucial role in determining therapeutic effects in glioma and the prognosis of patients with glioma (49, 50). The interaction between glioma cells and macrophages could promote the proliferation and invasion of tumor cells (51), and Weenink et al., found that the level of CD8+ T cells in glioma was positively correlated with poor prognosis (52). Therefore, we also explored the relationship between immune cell infiltration and risk scores in the prognostic risk model and found that risk scores were correlated, to some extent, with immune cell infiltration.

Previous studies have also demonstrated that the expressions of immune checkpoint and HLA complex are an important factor in determining the response to treatment and the prognosis of patients with glioma (53–55). Lemke et al. investigated the role of B7-H3 in glioblastoma and elucidated its mechanism in the most fatal type of glioblastoma (56), and Machulla et al. found that the expression of HLA was positively correlated with the occurrence of gliomas (57). We also found that the risk scores were positively correlated with the expressions of B7-H3, CTLA4, LAG3, PD-1, PD-L1, PD-L2, TIM-3, HLA-A, HLA-B, and HLA-C. These results confirm the reliability of the model in predicting the immune microenvironment of glioma.

There are two main limitations of the present study. Firstly, clinical information downloaded from TCGA and CGGA databases is limited and incomplete, and secondly, the identified mechanism of ARGs affecting glioma development requires further *in vivo* and *in vitro* experimental studies.

In summary, we used 15 ARGs to construct a prognostic risk model that accurately predicted the prognosis of glioma patients. The risk score generated by this model can be used as an independent prognostic indicator to discriminate patients with different survival outcomes. The model can also predict the immune microenvironment of glioma to a certain extent. However, further studies are needed using a prospective, large-scale, multicenter clinical cohort to validate the prognostic model.

## Data Availability Statement

Publicly available datasets were analyzed in this study. This data can be found here: https://portal.gdc.cancer.gov/, http://www.cgga.org.cn/.

## Author Contributions

CZ, KY, and RL conceived and designed the study, edited the manuscript, and acted as overall guarantors. YX and RL carried out the analysis procedure, contributed to the study design and interpretation of the results, and contributed to the drafting of the manuscript. XL, ND, DW, and LH contributed to the preparation of the dataset and drafts of the manuscript. All the authors reviewed the manuscript.

## Conflict of Interest

The authors declare that the research was conducted in the absence of any commercial or financial relationships that could be construed as a potential conflict of interest.

## Footnotes

1 https://tcga-data.nci.nih.gov/tcga/

2 http://www.cgga.org.cn/

3 http://www.autophagy.lu/

4 http://www.proteinatlas.org
