# Supplementary material for: An Autophagy-Related Gene Signature Associated With Clinical Prognosis and Immune Microenvironment in Gliomas
Source: Front Oncol. 2020 Oct 19;10:571189. doi: 10.3389/fonc.2020.571189 (PMC7604433; doi:10.3389/fonc.2020.571189)
Supplement: Supplementary file 1 [file Table_1.DOCX]

Supplementary Material

# Supplementary Tables and Figures

**1.1** Supplementary Tables

**Table S1**. Demographics and Clinical characteristics of glioma patients in the TCGA cohort and

| Variables | TCGA | | |  | CGGA | | |
| --- | --- | --- | --- | --- | --- | --- | --- |
|  | Total（n=665） | Low risk（n=333） | High risk（n=332） |  | Total（n=929） | Low risk（n=464） | High risk（n=465） |
| Grade |  |  |  |  |  |  |  |
| WHO II | 214 | 173 | 41 |  | 270 | 193 | 77 |
| WHO III | 236 | 122 | 114 |  | 305 | 180 | 125 |
| WHO IV | 159 | 1 | 158 |  | 350 | 89 | 261 |
| NA | 56 | 37 | 19 |  | 4 | 2 | 2 |
| Age |  |  |  |  |  |  |  |
| <65 years | 517 | 280 | 237 |  | 887 | 459 | 428 |
| ≥65 years | 92 | 16 | 76 |  | 41 | 4 | 37 |
| NA | 56 | 37 | 19 |  | 1 | 1 | — |
| Gender |  |  |  |  |  |  |  |
| Female | 254 | 126 | 128 |  | 380 | 199 | 181 |
| Male | 355 | 170 | 185 |  | 549 | 265 | 284 |
| NA | 56 | 37 | 19 |  | — | — | — |
| IDH status |  |  |  |  |  |  |  |
| Wild type | 236 | 10 | 226 |  | 400 | 67 | 333 |
| Mutant | 420 | 321 | 99 |  | 484 | 369 | 115 |
| NA | 9 | 2 | 7 |  | 45 | 28 | 17 |
| 1p 19q status |  |  |  |  |  |  |  |
| Non-codeletion | 495 | 190 | 305 |  | 667 | 266 | 401 |
| Codeletion | 165 | 143 | 22 |  | 191 | 162 | 29 |
| NA | 5 | — | 5 |  | 71 | 36 | 35 |

CGGA cohorts based on the autophagy signature.

NA, not available.

**1.2** Supplementary Figures


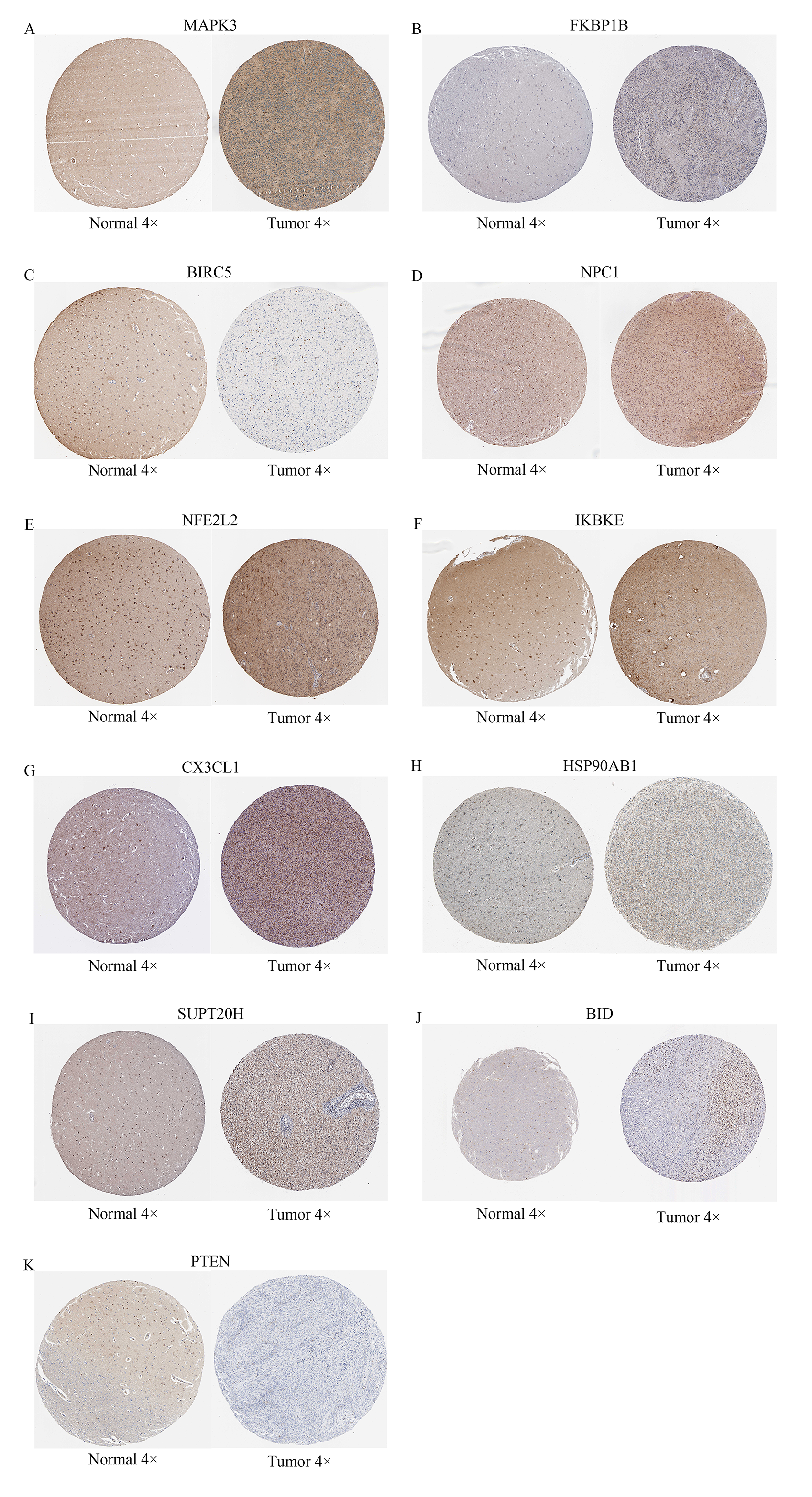


**Figure S1.** Expression and survival analysis for risk genes in glioma. The expression profiles of the proteins encoded by MAPK3(A), FKBP1B(B), BIRC5(C), NPC1(D), NFE2L2(E), IKBKE(F), CX3CL1(G), HSP90AB1(H), SUPT20H(I), BID(J) and PTEN(K) in normal and tumor tissues using clinical specimens from the Human Protein Profiles (DAPK2, MAPK8IP1, GABARAP and PKCQ are not found)..


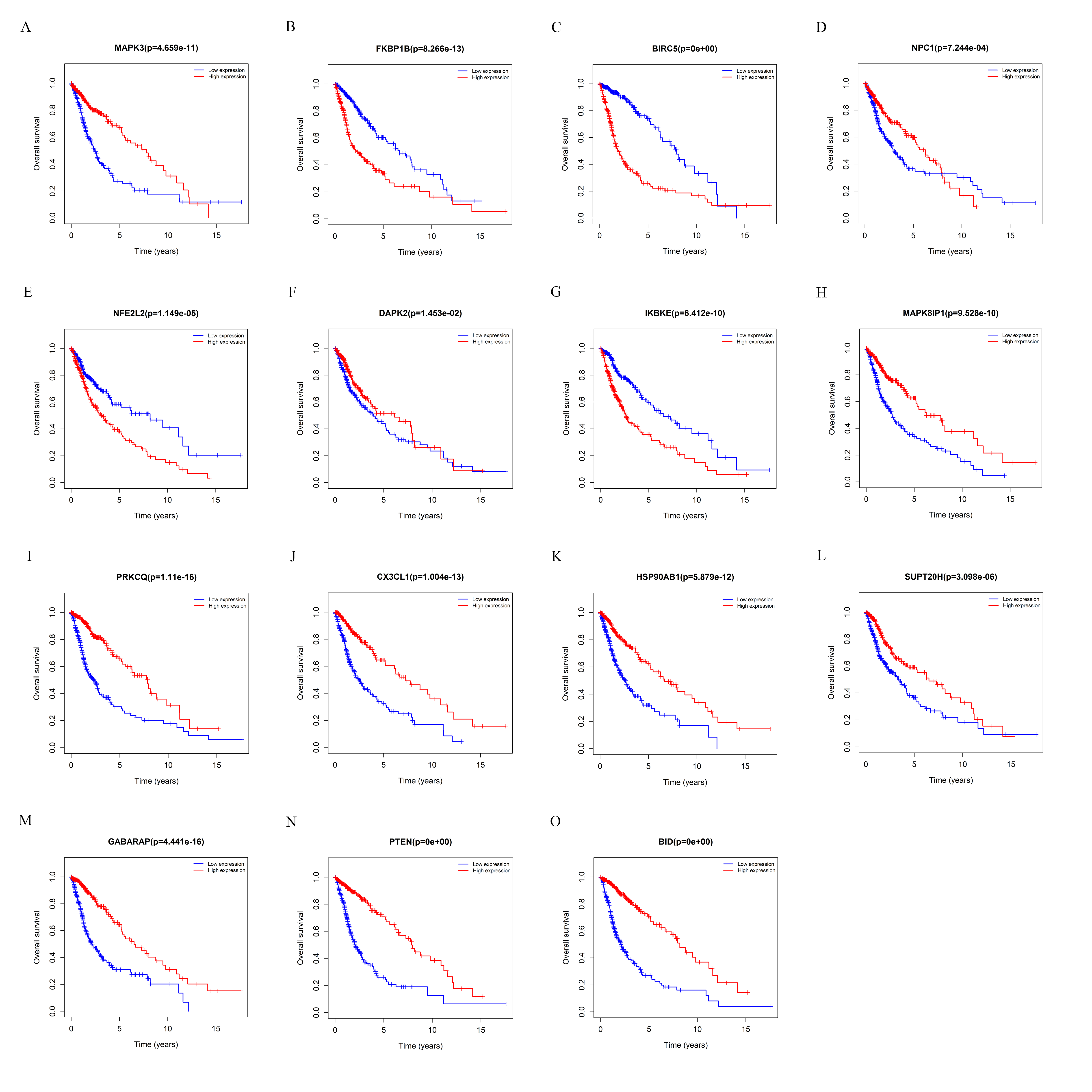


**Figure S2.** Survival analysis for risk genes in glioma**.** K-M OS curves based on the expression levels of MAPK3(A), FKBP1B(B), BIRC5(C), NPC1(D), NFE2L2(E), DAPK2(F), IKBKE(G), MAPK8IP1(H), PRKCQ(I), CX3CL1(J), HSP90AB1(K), SUPT20H(L), GABARAP(M), BID(N) and PTEN(O) in patients with glioma in the TCGA dataset**.**

**
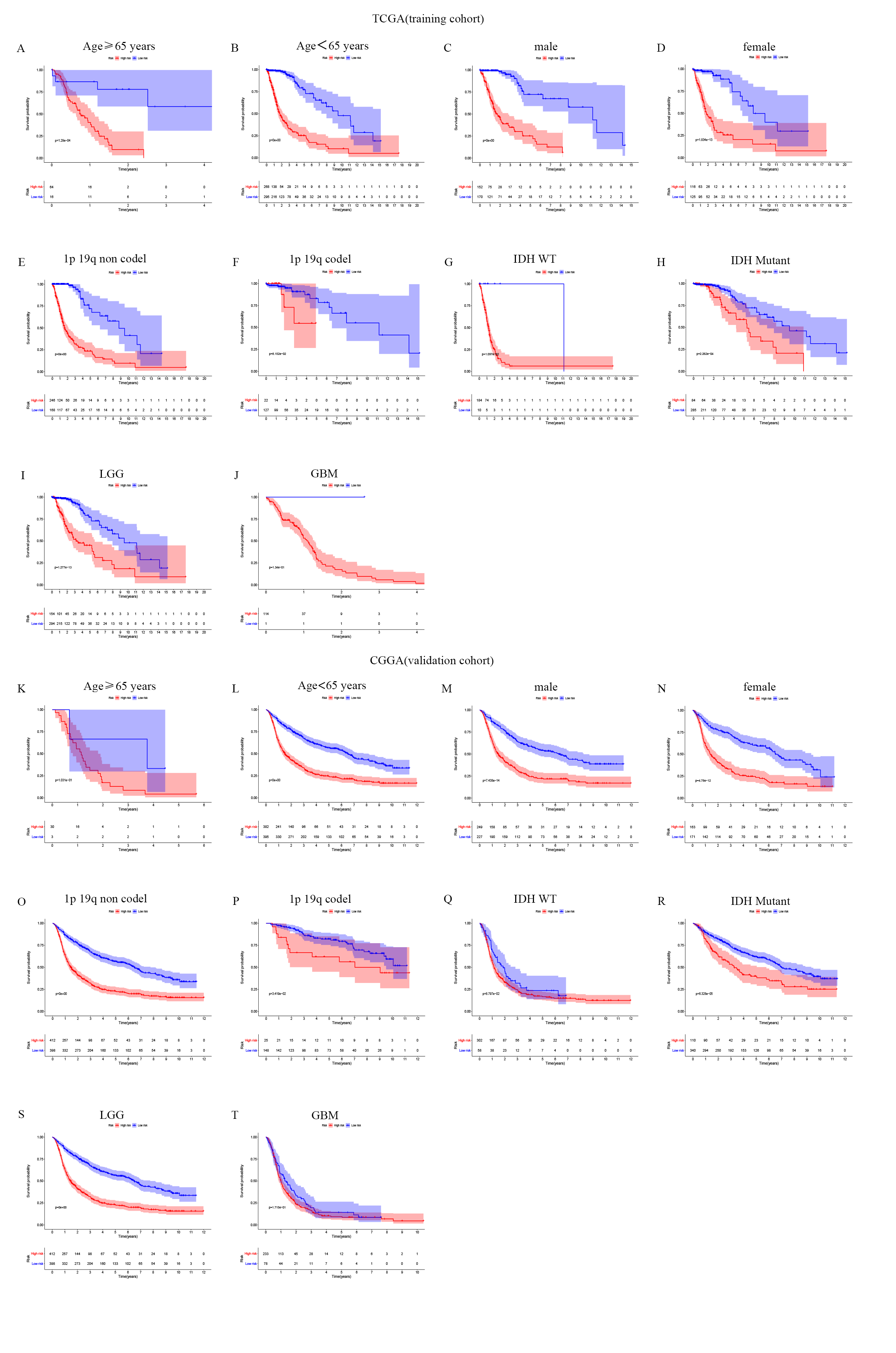
**

**Figure S3** Kaplan-Meier survival curves of OS according to low- or high-risk scores stratified by age, gender, 1p 19q codeletion status, IDH mutation status and WHO grade in the TCGA training cohort (A-J), CGGA validation cohort (B-U).


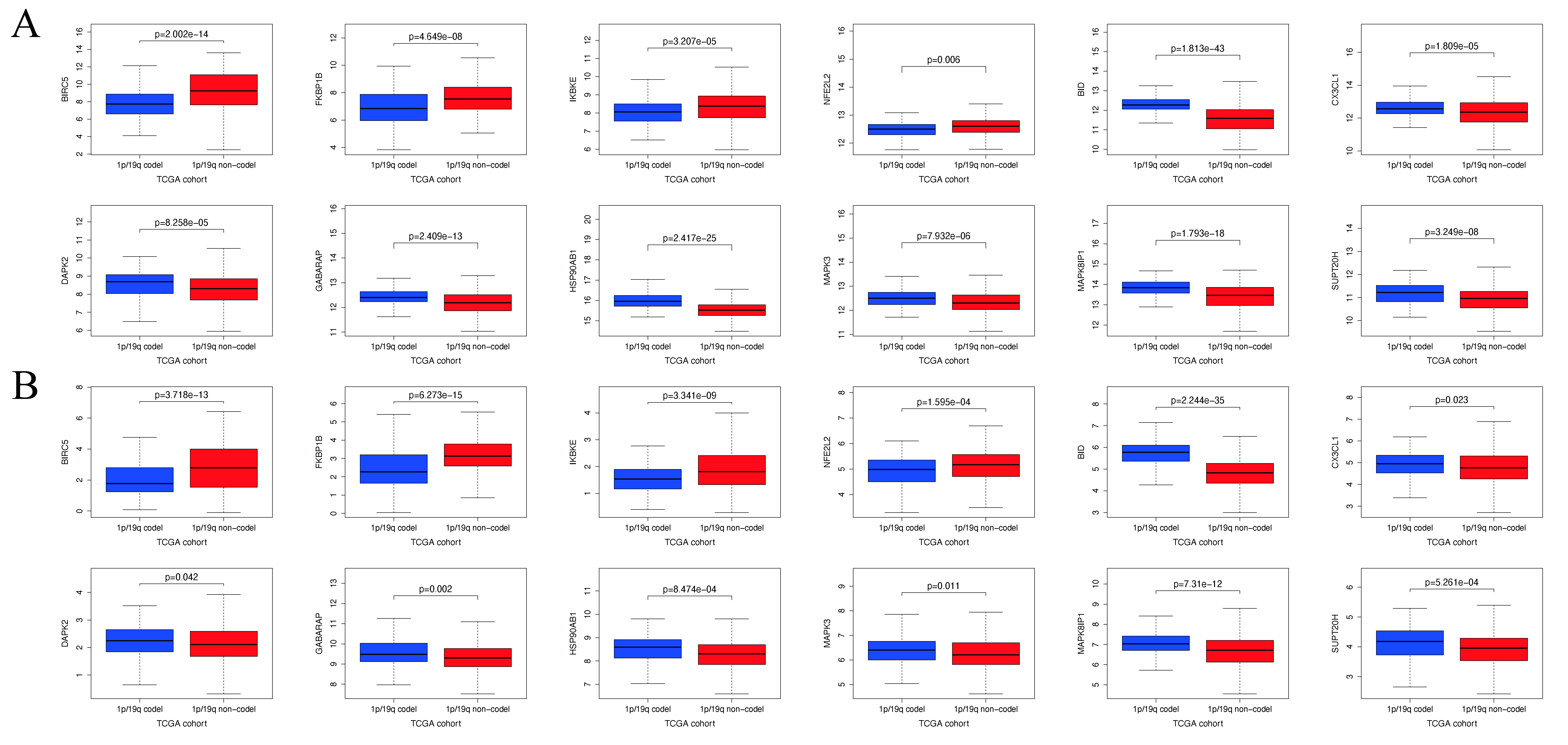


**Figure S4.** Relationships of the risk genes expressions with the 1p/19q codeletion status of glioma in the TCGA (A) and CGGA cohorts (B).


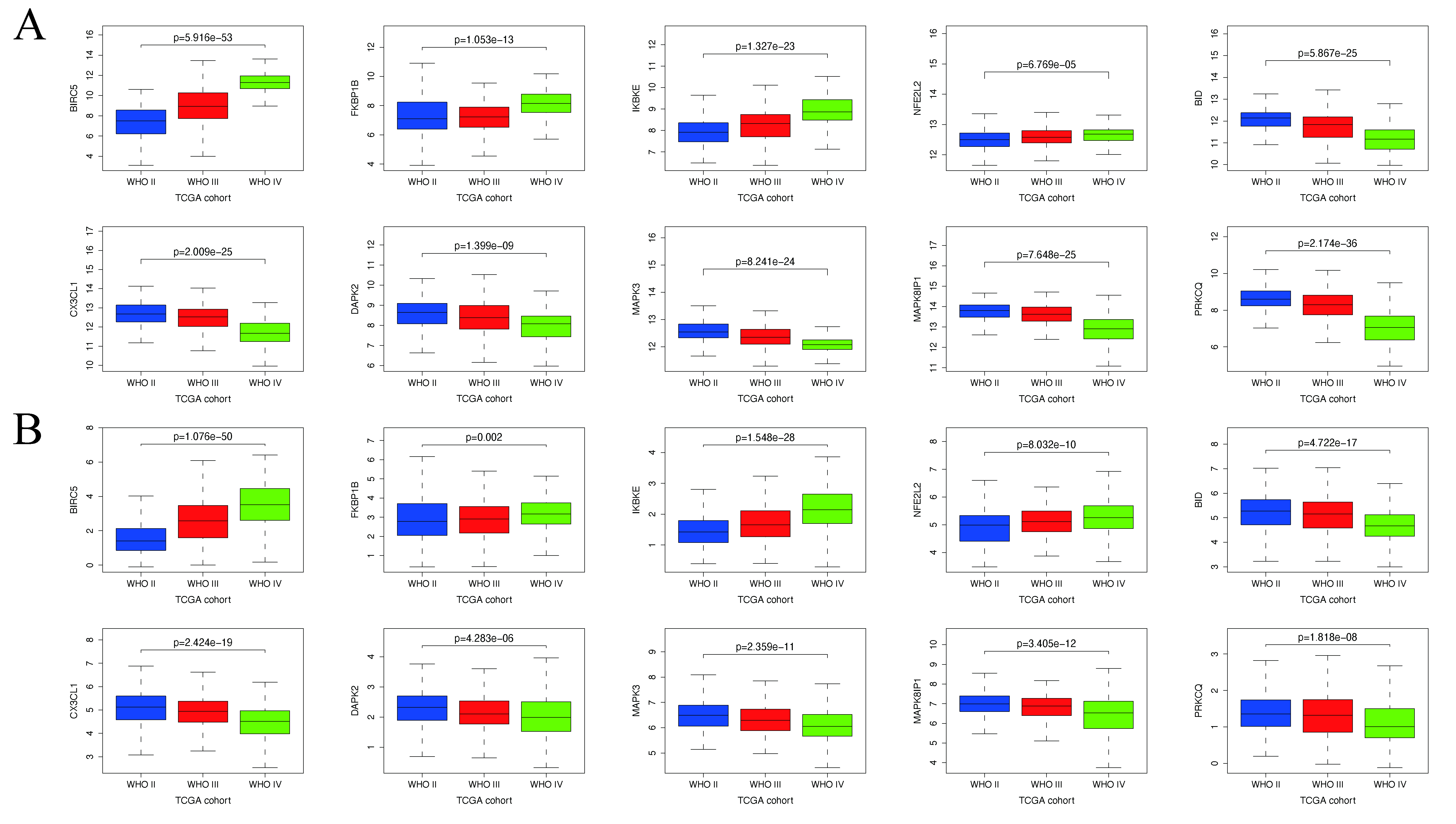


**Figure S5.** Relationships of the risk genes expressions with the WHO grade of glioma in the TCGA and CGGA cohorts.

**
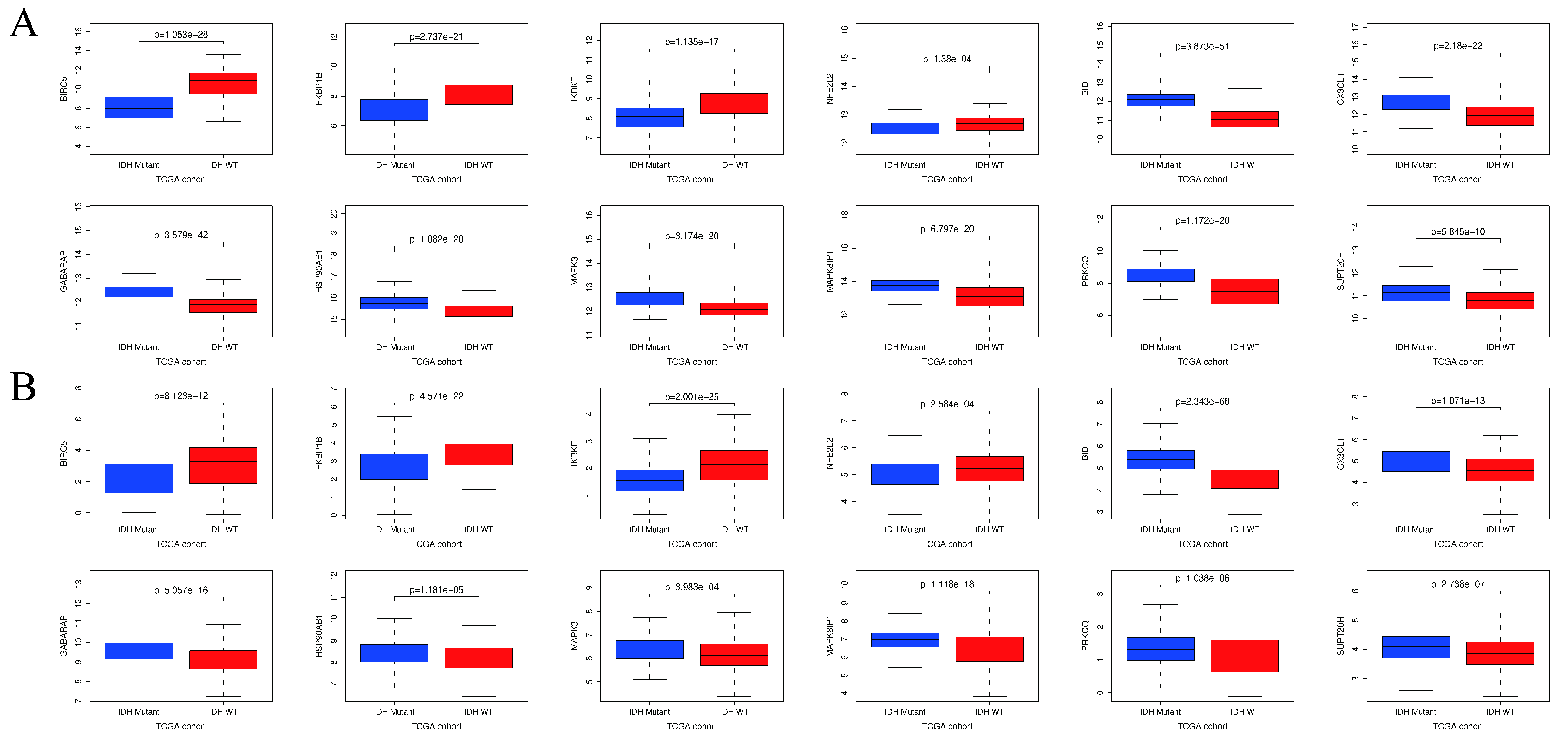
**

**Figure S6.** Relationships of the risk genes expressions with the IDH mutation status of glioma in the TCGA and CGGA cohorts.
